# SUPRACONDYLAR FRACTURES IN CHILDREN: A SYSTEMATIC REVIEW OF TREATMENT OPTIONS

**DOI:** 10.1590/1413-785220243203e278420

**Published:** 2024-08-02

**Authors:** Douglas Hideo Higuchi, Gabriel Alencar de Oliveira, João Paulo Alves, Lucas Lebedenco, Eiffel Tsuyoshi Dobashi

**Affiliations:** 1 Universidade Federal de São Paulo – UNIFESP, Escola Paulista de Medicina, Departamento de Ortopedia e Traumatologia, São Paulo, SP, Brasil.

**Keywords:** Humeral Fractures, Child, Fracture Fixation, Orthopaedic Fixation Devices, Postoperative Complications, Fraturas do Úmero, Criança, Fixação de Fratura, Dispositivos de Fixação Ortopédica, Complicações Pós-Operatórias

## Abstract

Objective: To compare the outcomes of surgical stabilization of pediatric supracondylar humeral fractures with the use of crossed Kirschner wires versus divergent lateral pinning wires. Methods: This is a systematic review with meta-analysis carried out by searching the MEDLINE/PubMed, Science Direct and Scielo databases. In these, the search for journals was carried out between January and August 2023, where 695 studies were found. To assess the quality of the studies, the Jadad and the MINORS scales were used.. The selection and reading of relevant articles were carried out by the researchers and 11 studies met the selection criteria. Results: From the 11 selected studies, 963 patients who met the criteria for the surgical treatment of these fractures were grouped. After the statistical analysis, we found that the ulnar nerve injury had a higher incidence when the crossed-K wire technique was used; and the lateral fixation is safer for the ulnar nerve. Conclusion: Both fixation techniques determine good functional results. However, fixation with lateral Kirschner wires proves to be safer considering the risk of iatrogenic injury to the ulnar nerve. Crossed-K wire fixation is more effective in terms of stability and maintenance of fracture reduction. **Level of Evidence II, Systematic Review of Level II or Level I Studies with discrepant results.**

## RESUMO

*Objetivo: Comparar os desfechos da estabilização cirúrgica das fraturas supracondilianas do úmero pediátricas com o uso de fios de Kirschner cruzados versus fios laterais divergentes. Métodos: Trata-se de uma Revisão Sistemática com metanálise realizada pela pesquisa nas bases de dados MEDLINE/PubMed, Science Direct e Scielo. Nessas, a busca dos periódicos foi realizada entre janeiro e agosto de 2023, e foram encontrados 695 estudos. Para a avaliação da sua qualidade, foram utilizadas a escala de Jadad e a escala MINORS. A seleção e a leitura dos artigos pertinentes foram realizadas pelos pesquisadores e 11 estudos preencheram os critérios de escolha. Resultados: Dos estudos selecionados, agrupamos 963 pacientes que preenchiam os critérios para o tratamento cirúrgico das fraturas. Após a análise estatística, observamos que maior incidência de lesão do nervo ulnar quando foi utilizada a técnica de pinagem cruzada; e a fixação lateral demonstrou ser mais segura para tal. Conclusão: Ambas as técnicas de fixação determinam bons resultados funcionais. Entretanto, a fixação com fios de Kirschner laterais demonstra ser mais segura, considerando o risco de lesão iatrogênica do nervo ulnar. A fixação com fios cruzados é mais eficaz, levando em conta a estabilidade e a manutenção da redução das fraturas.* **Nível de evidência II, Revisão sistemática de Estudos de Nível II ou Nível I com resultados discrepantes.**

## INTRODUCTION

 Supracondylar fractures of the humerus (SFH) account for about 3 to 15% of all bone lesions affecting the immature skeleton, especially in children under seven years of age. ^1^ It is the fracture that most requires surgical treatment in the pediatric population, ^2^ with an estimated incidence of 1.7 per 1,000 individuals. ^3^

 In general, these lesions are treated by closed reduction associated with percutaneous fixation with Kirschner wires (KW). This osteosynthesis method offers several configurations that can arrange implants in various ways, typically by cross (two lateral and one medial or one medial and one lateral wires) or lateral entries (three or two divergent or two parallel wires). ^4^

 Successfully treating pediatric SFH depends on achieving and maintaining an acceptable reduction until the fracture consolidates itself, avoiding potential complications. ^5^

 Therapeutic advances and improvements in the care of SFH have undoubtedly contributed to the success of the treatment (which depends on obtaining and maintaining an adequate and stable reduction until the fracture consolidates itself). ^6^

 The possible complications of these fractures especially include nerve and vascular injuries, compartment syndrome, malunion, and functional impairment (including reduced range of motion and angular deformities). ^1^ , ^5^

 Controversy persists regarding the choice of the ideal fixation technique for these fractures. Although the literature describes many pin configurations with KW, the two most common refer to cross-fixation and osteosynthesis with a lateral entry. However, despite its many articles, this review acknowledges the persisting controversies on this topic. ^7^

Based on this problem, the authors of this study aim to analyze the existing literature, carry out a secondary systematic review with a meta-analysis, and compare the efficiency of several configurations of osteosynthesis with KW regarding their stability and reduction of complications in pediatric SFH.

## METHODOLOGY

 This systematic review was carried out with a targeted protocol using the Preferred Reporting Items for Systematic Reviews and Meta-Analyses (PRISMA). ^8^

Primary cross-sectional, cohort, and randomized studies and case reports on the use of wires in children with SFH in all languages that were published in the last 10 years were considered as inclusion criteria.

 The guiding question of this research followed the PICO strategy (P – population, I – intervention, C – comparison, and O – outcome). ^8^ Its study population consisted of children with SFH; its intervention, of osteosynthesis with cross-arranged KW in comparison to other techniques (such as lateral fixation); and its outcome, of consolidation, function, and complication rates. Thus, this study elaborated the following clinical question: “What fixation technique for supracondylar fractures offers the best stability and complication rates?” 

 Searches were conducted from January to August 2023 on the following databases: Medical Literature Analysis and Retrieval System Online/National Library of Medicine (MEDLINE ^®^ /PubMed ^®^ ), Science Direct, and Scientific Electronic Library Online. Additional searches were performed on the reference list of the studies of interest to refine the search and include research that had been missed. 

The search strategy in this study considered descriptors that were selected from the DeCS/MeSH (Descritores em Ciências da Saúde/Medical Subject Headings) in Portuguese and English, which were combined by the Boolean operator AND: “distal humeral fractures” AND “fracture fixation” AND “child” or “humeral fractures, distal” AND “fracture fixation” AND “children.”

All retrieved studies were independently evaluated by two authors, who screened them by reading their titles and abstracts. Potentially eligible texts were reviewed and then fully read. Disagreements regarding article choice were solved by a discussion among the involved researchers. However, a third author was consulted to resolve possible discrepancies, whenever necessary.

 The quality of the trials was assessed by the Jadad scale ^9^ for randomized clinical trials and by the Methodological index for non-randomized studies (MINORS), ^10^ for observational studies. 

The data collected during the search were detailed in a spreadsheet in which all the information was made available as tables.

## RESULTS

**Figure 1.**
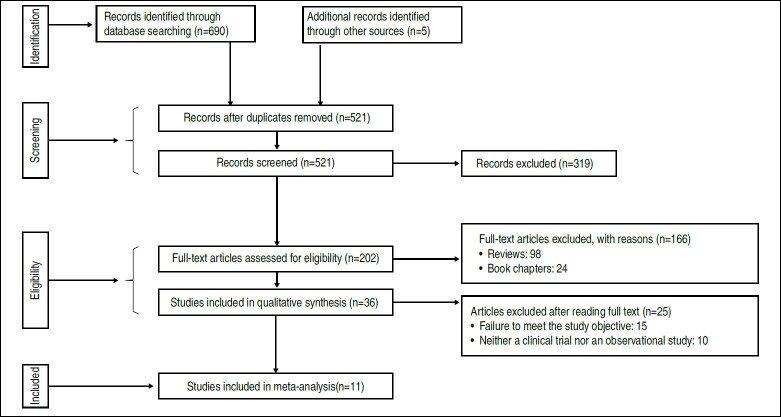
Diagrama de fluxo conforme recomendação PRISMA.

 Of the 695 retrieved studies, this research excluded 659 for failing to meet its pre-established inclusion criteria or for being duplicates. Thus, 36 studies underwent a detailed analysis. Finally, the final evaluation included 11 clinical studies: nine from electronic searches, ^11^ - ^19^ and two from manual searches of the references of other articles. ^20^ - ^21^ Figure 1 details the process of sorting the articles in a flow diagram. 

 According to Table 1 , only Afaque et al. ^12^ showed good methodological quality in the Jadad scale. ^9^

**Table 1.** Jadad ^9^ evaluation of the studies.

Jadad Evaluation. ^9^	Afaque et al. ^7^	Jain et al. ^11^	Natalin et al. ^12^	Othman et al. ^13^
Was the study described as randomized?	1	0	0	0
Was randomization described and was it adequate?	1	1	1	1
Was the study described as double-blind?	0	0	0	0
Was blinding described and was it appropriate?	0	0	0	0
Were losses and exclusions been described?	1	1	1	1
Total:	3	2	2	2

≥ 3: low risk of bias; < 3: High risk of bias.

 Source: Jadad et al. ^9^

 Items on the MINORS ^10^ scale are rated as 0 (unreported), 1 (reported but inadequate), or 2 (reported and adequate). This analysis showed that Claireaux et al. ^11^ and Trung et al. ^18^ obtained the lowest scores ( Table 2 ). 

**Table 2.** Classification of study quality and risk of bias according to the MINORS ^21^ tool.

Author/ Year	Study follow-up time	MINORS Score ^21^
Claireaux et al. ^10^	24 weeks	6
Yawar et al. ^14^	24 weeks	9
Rutuarama and Firth ^15^	24 weeks	12
Trung et al. ^16^	48 weeks	6
Agrawal et al. ^17^	24 weeks	10
Moratelli et al. ^18^	8 weeks	10
Li et al. ^19^	60 weeks	11

 Source: Slim et al. ^10^

Table 3 lists the general characteristics of the studies in this systematic review.

**Table 3.** Summary of the studies in this systematic review.

Author/ Year	Sample	Type of Fracture	Intervention	Follow-up (weeks)	Outcome
Claireaux et al. ^10^	N: 209 patients	52.0% girls	6.4 years on average	Gartland Type II or III	Different diameters	Kirschner crossed wires	24	Significant loss of reduction and neurological deficit were observed.
Afaque et al. ^7^	N: 84 patients	70.0% boys	6.8 years on average	Gartland Type III	Crossed fixation	Lateral fixation	12	Both techniques provided stable fixation and good functional results without iatrogenic injuries to the ulnar nerve.
Jain et al. ^11^	N: 168 patients	70.2% boys	6.8 years on average	Gartland Type III	Crossed fixation	Lateral fixation	24	Both groups showed a significant loss of range of motion and the cross-fixation group, iatrogenic nerve injuries.
Natalin et al. ^12^	N: 43	65.0% boys	6.5 years on average	Gartland Type III	Crossed fixation	Lateral fixation	8	Observed neither compartment syndrome, vascular or treatment-related nerve injuries nor pin infections.
Othman et al. ^13^	N: 47	Sex: unreported	5.5 years on average	Gartland Type II or III	Dorgan’s Cross Lateral Fixation	Medial-lateral cross fixation	Parallel or divergent side fixation	28	The aesthetic clinical result was satisfactory for the three techniques in more than 90% of the cases.
Yawar et al. ^14^	50 patients	52.0% boys	6.3 years on average	Gartland Type II or III	Crossed fixation	Lateral fixation	24	Both lateral and crossed wire configurations led to good radiological stability.
Rutuarama and Firth ^15^	N: 38	66.0% boys	7.5 years on average	Gartland Type III	Closed reduction and Kirschner crossed percutaneous wires	24	Most children regained full range of motion after closed reduction and fixation of crossed wires without physical therapy.
Trung et al. ^16^	N: 42	70.0% boys	6.0 years on average	Gartland Type II or III	Cross-pining technique with a Kirschner wire inserted medially and another laterally	48	Closed reduction and percutaneous fixation proved to be an effective treatment with good therapeutic results.
Agrawal et al. ^17^	N: 70	65.4% boys	8.0 years on average	Gartland Type II or III	Closed reduction and fixation by two Kirschner crossed wires.	24	Satisfactory functional results, brief hospital stays, and few complications of percutaneous fixation with Kirshner crossed wires.
Moratelli et al. ^18^	N: 129	59.7% boys	6.3 years on average	Gartland Type II or III	Crossed fixation	Lateral fixation	8	Lateral or cross fixation and time to surgery failed to influence the functional outcomes of supracondylar fracture in children but lateral fixation decreases the risk of ulnar nerve injuries.
Li et al. ^19^	N: 83	73.5% boys	10.0 years on average	Gartland Type III	Small medial approach and cross-fixation with three Kirschner wires.	60	Low incidence of complications in older children.

 The sample consisted of 963 children, of whom 453 underwent fixation using the cross-wire technique and 410, osteosynthesis with lateral wires or other techniques. Samples ranged from 38 ^15^ to 209 patients in the included studies. ^11^ About six articles included Gartland type II and III fractures, whereas five, only type III fractures. 

 Most studies showed a higher prevalence in boys, with percentages ranging from 52.0 to 73.5%. ^15^ - ^20^ Only one study had a higher prevalence of girls(52.0%). ^11^

 Except for one study, ^17^ which only used cross-KW, the distribution of fixation techniques ranged from 48.05 ^11^ to 66% ^14^ for lateral wires and from 34 ^15^ to 51.95% for cross-wire. ^12^

 Nerve injury incidence reached 11.11% at most in two studies ^12^ , ^13^ and that of infection in pin path, 31.20% ^22^ in one study and zero in another study. ^13^ Only one study ^21^ reported vascular injuries. 

All studies employed crossed KW or lateral fixation, with follow-ups ranging from eight to 60 weeks. All studies showed satisfactory results, regardless of osteosynthesis technique. Considering complications, this study highlights the risk of ulnar nerve injuries and loss of reduction.

**Figure 2.**
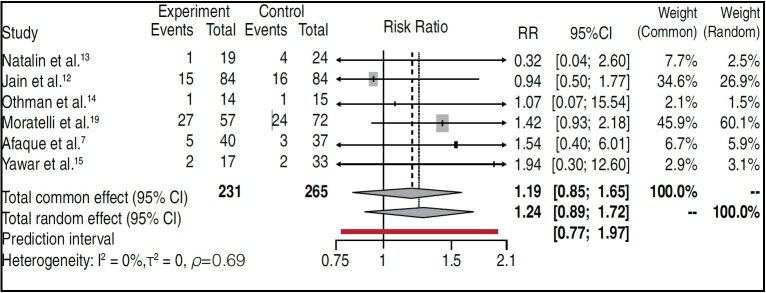
Forest plot showing the proportion of complications between cross and lateral fixation.

 Statistical analysis first carried out a meta-analysis with studies that included control groups. ^7^ , ^12^ - ^15^ , ^19^ This model used a meta-analysis of binary outcomes (occurrence or absence of complications in both groups and crossed or side wires). This research considered both common and randomized effects and returned the hazard ratio to compare the chosen studies. The results of this meta-analysis ( Figure 2 ) indicated no differences between the complications in the group that received cross-fixation and that which received lateral fixation (RR 1.19 and 1.24; CI-0.77; 1.9; p = 0.69) and no evidence of heterogeneity between studies (I ^2^ = 0%, τ ^2^ = 0). 

**Figure 3.**
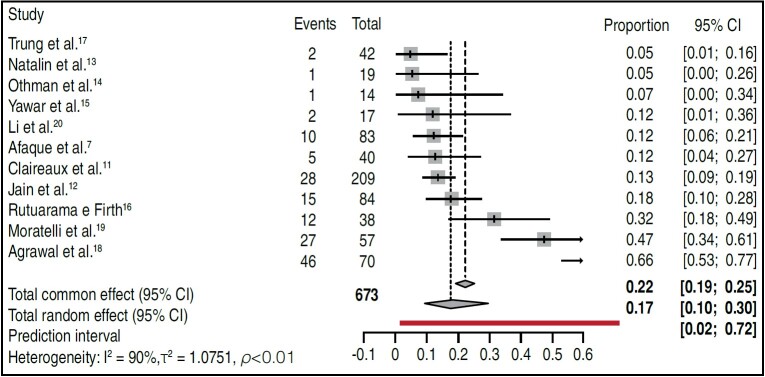
Forest plot showing the proportion of complications in the included studies.

 The second meta-analysis used individual proportions by combining the proportions or probabilities of an event across studies to calculate an overall proportion or probability. The results of this meta-analysis ( Figure 3 ) indicated differences between the complications in each study (RR 0.22 and 0.17; CI −0.02; 0,72; p = 0.01) and a high evidence of heterogeneity between studies (I ^2^ = 90%, τ ^2^ = 1,0751). 

## DISCUSSION

The successful treatment of SFH with deviations depends on maintaining an acceptable reduction until the fracture heals, avoiding complications.

Controversy persists regarding the optimal technique of fixation with KW. So, this systematic review was carried out to try to ascertain the most efficient surgical technique of osteosynthesis.

 The analysis of the stability of several fixation configurations retrieved the biomechanical study by Zionts et al., ^23^ who measured the rotational resistance of the SFH distal fragment by simulating and fixating them in four configurations. The authors concluded that the configuration of crossed wires placed from the medial and lateral condyles configured the arrangement with the greatest mechanical stability. However, two parallel lateral KW could serve to treat significant swelling of the upper limb to be operated despite it being an inferior but acceptable biomechanical option. More recently, Lee et al. ^3^ used a bone model and concluded that the use of two divergent lateral pins was comparable to crossed wires in extension and varus and valgus loading but would be biomechanically inferior in axial rotation tests. Stability using parallel or divergent lateral fixation can be improved by maximally separating the pins at the fracture site and adding a third pin in the middle in cases of significant movement at the fracture focus. This review considers that inserting KW through the olecranon fossa adds two more cortices, increasing stability. ^5^

 Other biomechanical studies show that the medial portion of the distal humerus suffers from greater stress and deformation under axial loads than the middle portion. Therefore, the internal rotation of the distal fragment is considered the main factor for varus deformity. ^4^ Therefore, adequate reduction and stable fixation should be achieved to avoid distal fragment deviation and postoperative deformity. ^14^ The displacement of SFH is more likely to occur in older children, as shown by some studies. ^22^

The most significant findings of this study, considering stabilization with crossed wires, refer to its higher risk of infection and ulnar nerve injuries. However, this technique has greater biomechanical stability. Lateral fixation offers a greater risk of loss of reduction.

 These findings resemble those in a systematic review with a meta-analysis by Kwok et al. (which included 11 studies), which reported that lateral fixation is associated with greater loss of reduction and lower risk of iatrogenic ulnar nerve injuries. ^6^

 However, this review deems that these findings should be carefully interpreted due to the poor methodological quality of most of the included studies and the divergent opinions on this topic. This study found a systematic review with a meta-analysis of randomized clinical trials that aimed to evaluate SFH stabilization techniques considering elbow function, risk of neurological injury, and loss of reduction. It evaluated results for certain aspects that resemble those in this study. It should be noted that this review included studies with a poor methodological quality. ^4^

 Claireaux et al. ^11^ found no significant differences in the incidence of neurological deficits and iatrogenic nerve injuries related to the care of patients treated with cross-sectional KW and other techniques. However, they observed that the maintenance of the reduction was significantly better in patients treated with the three Kirschner-wire configuration (two lateral and one medial) than those under other configurations. Moreover, patients treated in this way showed a smaller change in Baumann angle. 

 Similarly, according to Natalin et al., ^13^ 56.0% of patients received fixation with lateral wires and 44.0%, with crossed wires. Overall, four had neurological injuries in their first consultation (which completely and spontaneously regressed during follow-up). Observed neither compartment syndrome, vascular or treatment-related nerve injuries nor pin infections. The authors also found that the elbow flexion amplitude decreased in the group of patients who underwent fixation with crossed wires, but no change in the Baumann angle between the different types of fixation. 

 Afaque et al. ^7^ also compared cross and lateral fixation and observed no differences regarding radiographic and clinical results between groups. Overall, two patients who underwent cross-fixation developed tardy ulnar nerve palsy. However, after statistical analysis, both techniques provided stable fixation, union, and good functional results without iatrogenic injuries to the ulnar nerve after small incisions were performed to find the medial epicondyle. 

Queiroz et al.’s systematic review showed that percutaneous fixation with lateral wires for type II and crossed wires for type III and IV fractures associated with a minimal medial approach to protect the ulnar nerve would offer significantly lower chances of iatrogenic nerve injuries. The longer duration of the procedure configures a disadvantage of medial surgery but fails to discourage this approach due to its greater stability.

 On the other hand, Moratelli et al. ^19^ stated that KW medial fixation increases the risk of ulnar nerve injuries. 

 Othman et al. ^14^ evaluated Dorgan’s lateral cross fixation by osteosynthesis with medial and lateral cross fixation associated with parallel or divergent lateral fixation. They observed that all three methods stabilized the fracture and maintained reduction. However, lateral pins are safer for the ulnar nerve than medial pins. 

 However, the results by Jain et al. ^11^ and Moratelli et al. ^19^ suggest that the cross-fixation method is better than the lateral fixation method. These authors considered biomechanical stability, which avoids secondary angular deviation and the resulting vicious consolidation. However, they mention that the lateral fixation method may be safer as it shows no risk of injury to the ulnar nerve. 

 Similarly, Yawar et al. ^15^ found that lateral and crossed wire configurations led to good radiographic stability, preserving the Baumann angle without any loss of reduction or risk of iatrogenic nerve injuries. 

 Rutuarama and Firth’s ^16^ findings indicate that most children with grade III Gartland SFH completely recovered their elbow range of motion and had good functional results 24 weeks after closed reduction and fixation with percutaneous crossed KW. On the other hand, older children or those with associated neurovascular and soft tissue lesions had poor functional results. Corroborating these findings, Li et al. ^20^ found that open reduction by a medial approach and cross-fixation with three KW for severely displaced type III Gartland fractures is safe and effective, with a low incidence of complications in older children. 

 Agrawal et al. ^18^ found that infection in the pin path (31.2%) and pin malposition (27.8%) were the most frequent complications. However, after wire removal, a daily periodic dressing and the use of appropriate oral antibiotic therapy helped treat infections. Trung et al. ^17^ reported that some patients had secondary osteomyelitis and iatrogenic injuries of the ulnar nerve due to cross-pinning. According to Moratelli et al. ^19^ loss of reduction (3.9%) and iatrogenic ulnar nerve palsy (2.3%) occurred after fixation with crossed pins 

Iatrogenic nerve injuries may stem from local irritation, pressure, twisting or penetration of the medial pin, iatrogenic constriction of the cubital tunnel by a medial pin, and nerve transection.

 Thus, some surgical techniques can reduce the rates of ulnar nerve injury associated with medial fixation. Initially, inserting the lateral pin enables elbow extension to a flexion below 90° so that the ulnar nerve can be displaced in a more posterior direction before the insertion of the medial pin. A small incision over the medial epicondyle serves to isolate the ulnar nerve, especially under pronounced swelling. ^6^ , ^14^ , ^20^ , ^24^ Ultrasound-guided intervention and intraoperative nerve monitoring ^13^ - ^15^ are also mentioned as options. 

However, this review stresses that the analysis the overall probability of iatrogenic nerve injuries (including the radial and median nerves) shows an about 2% probability of neural damage even under a lateral entry point for KW affixation. This can occur due to the reduction maneuver and the penetration of the pins through the medial or anterior cortex.

This study has a number of limitations, some of which are inherent to all systematic reviews. The studies in this review show methodological variations, including fixation techniques between and in studies; various institutions, and surgeons’ particularities. Clearly defined technical guidance and precise adherence to certain principles, such as making a small incision and ensuring that a medial KW is inserted directly into the bone, can determine the lowest likelihood of iatrogenic nerve injuries. Careful placement of lateral entry pins, proper fixation of all three columns, bicortical fixation, and fluoroscopy can provide a lower rate of fracture displacement after fixation. Most included studies used retrospective case series with weaker empirical evidence than randomized controlled trials or prospective studies. Moreover, improvements in surgical techniques and radiographic technology likely affected results. The limited number of studies with a greater degree of scientific relevance negatively affected this research.

 This review agrees with Avenkar et al. ^21^ and Wang et al. ^24^ who stress the common frequency of complications after SFH in children. Moreover, Rutuarama and Firth ^16^ emphasize that these fractures can cause physical disability in children due to such potential complications. 

## CONCLUSION

The articles this review analyzed and included showed that both cross and lateral fixation techniques provide good functional results but fixation with lateral wires more safely avoided ulnar nerve injuries, whereas fixation with crossed wires more effectively maintained this reduction, conferring greater stability in infantile SFH.

Despite the findings of this study, the definition of the best method of fixation of these fractures in children (whether with crossed or lateral KW) remains uncertain. Thus, this review stresses the need for more randomized clinical trials to analyze the EXISTING osteosynthesis techniques and determine the best treatment for these fractures. Level of Evidence I, systematic review.
